# Systematic analysis of feeding behaviors and their effects on feed efficiency in Pekin ducks

**DOI:** 10.1186/s40104-017-0212-2

**Published:** 2017-11-01

**Authors:** Feng Zhu, Yahui Gao, Fangbin Lin, Jinping Hao, Fangxi Yang, Zhuocheng Hou

**Affiliations:** 1 National Engineering Laboratory for Animal Breeding and MOA Key Laboratory of Animal Genetics and Breeding, Department of Animal Genetics and Breeding, China Agricultural University, Beijing, 100193 China; 2 Beijing Golden Star Duck Inc., Beijing, 100076 China

**Keywords:** Economic traits, Feeding behavior, Feed efficiency, Pekin duck

## Abstract

**Background:**

Feeding behavior study is important for animal husbandry and production. However, few studies were conducted on the feeding behavior and their relationship with feeding efficiency in Pekin ducks. In order to investigate the feeding behavior and their relationship with feed efficiency and other economic traits in Pekin ducks, we selected 358 male Pekin ducks and recorded feeding information between 3 to 6 wk of age using automatic electronic feeders, and compared the feeding behavior under different residual feed intake (RFI) levels.

**Results:**

We observed that total feed time, daily feed intake and feed intake per meal had strong positive correlations with feed efficiency traits; moreover, strong correlation between feed intake per meal and body weight was found (*R*=0.32, 0.36). Daily feeding rate meal and meal duration had weak correlations with feed efficiency (*R*=0.14~0.15). The phenotypic correlation of between-meal pauses, with feed efficiency was not observed. When daily changes were analyzed, high RFI ducks had the highest feed consumption over all times, and obvious differences in daily visits were found among different RFI level animals during the middle period; these differences were magnified with age, but there was no difference in daily meal number. Moreover, our data indicate that high RFI birds mainly take their meals at the edge of the population enclosure, where they are more susceptible to environmental interference.

**Conclusions:**

Overall, this study suggests that the general feeding behaviors can be accurately measured using automatic electronic feeders and certain feeding behaviors in Pekin ducks are associated with improved feed efficiency.

**Electronic supplementary material:**

The online version of this article (10.1186/s40104-017-0212-2) contains supplementary material, which is available to authorized users.

## Background

Feed efficiency is a major economic trait of domestic animals. For most poultry, feed efficiency has been selected in variety of ways, such as feed conversion ratio (FCR) and residual feed intake (RFI) [1, 2]. Improvements in feed efficiency have been achieved by selecting for RFI [3, 4], and some studies have shown that FCR has the genetic potential to improve feed efficiency in Pekin ducks and mule ducks [5, 6]. New developed automatic recording machine helps us to account large number of individuals at the same time in breeding. In practice, we found ducks needs adaptation time at the beginning of using feeding machine. Adaptive learning ability was observed to be different in Pekin ducks at beginning of using machine. So learning animal feeding behavior would be important for animal husbandry, productions system design and animal welfare [7, 8].

It is widely recognized that behavior is an important aspect of the physiological status of animals [9]. Feeding behavior may reflect animal meal habit, as a potential predictor of feed efficiency [10]. Moreover, feeding behavior can also be used as an indicator for reflecting animal health situation. Early studies that utilized behavioral data depended on human observation or photographic information acquired by camera equipment. With the development of electronic feeders, individual feeding information can be collected automatically and measured accurately [11]. Recent studies observed the meal intervals showed considerable variations in Pekin ducks [12]. In broilers, feeding behaviors were found to be related with feed efficiency in different selected lines [13]. However, the general feeding behavior pattern is not clear and whether these feeding behavior affect feed efficiency in Pekin ducks is also unknown.

In order to investigate the relationship between feeding behavior and feed efficiency in Pekin ducks, this study selected 358 male Pekin ducks and recorded feeding information between 3 to 6 wk of age using electronic feeders. Our results provide basic feeding behavior data in Pekin ducks and found feeding behavior are strongly correlated with feed efficiency in Pekin ducks.

## Methods

### Birds and housing

All ducks were reared in the same house with the same feeding and lighting program. Each house hold 100 feeding machines. Ten machines were grouped together as one pen with barrier for duck management. We randomly selected 3 batches of 3 wk. Male Pekin ducks obtained from the Beijing Golden Star Company (358 birds in total). Each of the 3 batches (batch one: *n* = 126; batch two: *n* = 119; batch three: *n* = 113) was measured from 3 wk of age until 6 wk of age. The intervals between batches was 8 d and measurement was same for each batch. Ducks in each batch were fed ad libitum for 25 d in a single pen. The pen area was 96 m^2^ and contained 10 automatic feeders and a water tank. Illumination was 10 lx for 24 h during the entire period. The pen design is illustrated in Additional file 1: Figure S1.

### Feeding behavior data acquisition

We designed duck feeding machine which use RFID as sensor to record each duck. The design of duck feeding machine is similar with Bley and Bessei [12]. Feeding behaviors (entering time, exiting time, feed consumption) and live body were recorded for each feeding for each duck from 19 d to 42 d. Only one duck can enter the machine for each feeding event adjusting barrier according to duck body size. The machine has been extensively used in the duck breeding practices [14]. Each feeding machine was spaced at 0.5 m intervals, and the feeder ID was set from 1 to 10 in linear. The first machine was nearest the gate and the last one was against the barrier. Tiny electronic tags were permanently fastened to the upper neck of each duck until slaughtering, which couldn’t interfere with animal activity. Upon entering a feeder, each duck’s electronic label ID was recognized by the machine. The date and time at beginning and end of each visit was recorded. The central server collected the raw data from each feeder and calculated feed intake and meal duration. At the end of feeding, the ducks with very small body size, without measurement or unusual feeding behavior were removed before analysis of feeding behavior. Any visit that could not be assigned to a specific duck was removed from the data set before analysis. During the whole measuring period, 0.001% of missing record events were observed and these records were removed. Only the ducks with complete records were used for further analysis. Additionally, the records that individual weight were far outweighed other records on the same day were removed, which may be caused by interference from other ducks.

### Traits measurement

The body weight (BW) of each bird was automatically measured individually from the beginning to the end of feeding at each visit. Daily BW was calculated as the median of the BW measurement over the entire 24 h period of each day. To calculate feed efficiency, 19 d and 42 d BW was measured manually as beginning and end weight for the test period respectively. The BWG (body weight gain) and feed conversion ratio (FCR) were computed over the entire feeding period. The RFI for the feeding test period was calculated as the residual of the linear regression of the feed intake during the test. The regression equations shown below:

$$ \boldsymbol{Feed}\boldsymbol{Intake}=-\mathbf{1.1720}\kern0.5em +\kern0.5em \mathbf{1.8204}\kern0.5em \times \kern0.5em \boldsymbol{BWG}\kern0.5em +\kern0.5em \mathbf{17.9369}\kern0.5em \times \kern0.5em \left({{\boldsymbol{BW}}_{\mathbf{19}}}^{\mathbf{0.75}}\right)\kern0.5em +\kern0.5em \boldsymbol{RFI} $$

Based on the distribution of RFI, three subsets were selected for later analysis. Ducks in the bottom 10% of RFI were defined as the low-RFI level, from 45% to 55% were defined as the middle-RFI level and the top 10% RFI individuals were selected as the high-RFI group. Each subgroup had 33 ducks, and these ducks were randomly chosen from the combined data.

Every visit was treated as a valid record if feed intake and meal duration were greater than zero. The intervals between visits were calculated to estimate the meal criterion (Additional file 2: Figure S2). The meal criterion was estimated using the method 1 developed by Howie et al. [15]. For each duck, visits were defined as part of a meal if the interval between visits was lower than the meal criterion. Feeding behavior traits were calculated daily and over the test period for each duck: feed intake, feeding rates, average intervals between meals, number of visits and number of meals. The distribution of records was also analyzed to investigate the feeding tendency of ducks.

Because feeding patterns of animals are affected by the environment and their growth needs, the data has been divided into three stages, based on previous analysis. The first three days were considered as a period of adaptation, the fourth day to the tenth day after the commencement of the study was defined as the pre-growth period, and the eleventh to twentieth day was classified as the growth period.

### Data analysis

Data management and statistical analyses were performed using R software. The variance analysis for different RFI levels was performed based on mixed models using R package *VCA*. The following fixed effects, for which *P* < 0.05, were retained:

$$ {y}_{ij k}=\upmu \kern0.5em +\kern0.5em {batch}_i\kern0.5em +\kern0.5em {level}_j\kern0.5em +\kern0.5em {e}_{ij} $$

In formation, *level*_*j*_ was the RFI levels, and the *batch*_*i*_ was the batch effect. *E*_*ij*_ was the residual of the model. Additionally interaction effect between batch and RFI level was not significant in the data.

## Results

### Descriptive statistics and meal interval estimation

A total of 105,785 visits were recorded by the feeders. 28 ducks’ label were missing before slaughter. After filtering unqualified data, data from a total of 330 ducks were used in further analysis. We compared the batch effect for feed efficiency and feeding behavior. No significant batch effects were observed among three batches. In the following analysis, all three batch data were combined together for description analysis. The *R*-square correlation coefficient of regression of estimated feed intake was 0.42. The descriptive statistics of the measured traits are summarized in Table 1. During the test period, the average FCR was 2.7, average RFI was null by construction, with an SD of 0.6 kg. Moreover the average total feeding time during the test period was 320.6 min; the average number of meals per day was 8.6, and the average number of visits per day were 13.6. The average daily feed intake was 0.324 kg/d. The meal criterion was estimated to be 1,132 s. The frequency distribution of log_e_ - transformed intervals is illustrated in Additional file 2: Figure S2.

**Table 1:** Descriptive statistics of measured traits for Pekin ducks

Items		All (*n* = 358)			
		Mean	S.D.	Min	Max
General traits	BW 19, kg	1.1	0.09	0.74	1.4
	BW 42, kg	3.8	0.2	3.1	4.4
	BWG, kg	2.7	0.2	1.8	3.3
	Feed Intake, kg	7.2	0.8	3.9	9.4
	RFI, kg	0.0	0.6	−3.9	2.1
	FCR	2.7	0.3	1.3	3.4
Feeding behavior traits	Total Feed Time, min	320.6	72.9	204.9	616.6
	Daily feed intake, kg/d	0.324	0.037	0.234	0.669
	Meal Feed Intake, g	33.8	22.8	1.0	350.0
	Visit duration, s	76.6	20.0	31.0	149.3
	Meal duration, s	159.2	162.4	79.9	316.8
	Average interval between meals, min	161.8	123	91.0	309.5
	Daily Feeding Rate, g/min	20.3	3.7	14.0	34.1
	Number of meals per day	8.6	1.4	2	14.4
	Number of visits per day	13.6	3.1	8.9	28.8

BW 19: average body eight at d 19; BW 42: average body eight at d 42; RFI, residual feed intake; FCR, feed conversion ratio. S.D., standard deviation

### Phenotypic correlation

Table 2 shows the phenotypic correlation between feeding behavior and general traits. Total feeding time (TFT), daily feed intake (DFI), feed intake per meal (MFI) daily feeding rate (DFR) and meal duration (MD) had significant positive correlation with feed efficiency traits; moreover, significant correlation between feed intake per meal and body weights was found (*R* = 0.32, 0.36, respectively). However, for between-meal pauses (BMP), no strong phenotypic correlation was found with general traits (*P* > 0.05*)*.

**Table 2:** Phenotypic correlation between feeding and other traits

	Total feed time	Daily feed intake	Daily feeding rate	Between-meal pause	Meal feed intake	Meal duration	BW 19	BW 42	BWG
BW 19	0.08	0.33**	0.10	0.14*	0.36**	0.15**	1.00	0.44**	−0.07
BW 42	0.22**	0.62**	0.07	−0.13*	0.32**	0.1	0.36**	1.00	0.90**
BWG	0.20**	0.44**	0.03	−0.19**	0.11*	0.04	−0.07	0.9**	1.00
RFI	0.23**	0.58**	0.14*	−0.07	0.23**	0.12*	0.03	0.01	0.00
FCR	0.16**	0.45**	0.14*	0.06	0.27**	0.15**	0.44**	−0.13*	−0.34**
FI	0.31**	0.84**	0.15*	−0.12*	0.33**	0.16**	0.31**	0.65**	0.55**

BW 19: average body eight at d 19; BW 42: average body eight at d 42; RFI, residual feed intake; FCR, feed conversion ratio.; FI, feed intake; “**“*P* value < 0.01; ”*” *P* value < 0.05

### Comparison among different RFI level

Variance analysis for different RFI level is presented in Table 3. Unsurprisingly TFT, DFI and MFI was significantly different among different RFI levels (*P* < 0.05), because these feeding behavior traits are highly phenotypically correlated with feed efficiency (Table 2). For other feeding behavior traits, our results showed that there were slight differences among different RFI level ducks, but these were not significant.

**Table 3:** Comparison of measured traits among different RFI levels

Items		Low RFI(*n* = 33)		Middle RFI(*n* = 33)		High RFI(*n* = 33)	
		Mean	S.D.	Mean	S.D.	Mean	S.D.
General Traits	BW 19, kg	1.1	0.1	1.1	0.1	1.2	0.1
	BW 42, kg	3.9	0.3	3.8	0.2	3.9	0.2
	Feed intake, kg	6.5^c^	0.7	7.2^b^	0.4	8.4^a^	1.1
	RFI	−0.9^c^	0.59	−0.024^b^	0.027	0.9^a^	1.1
	FCR	2.3^b^	0.2	2.7^a^	0.1	2.7^a^	0.19
Feeding behavior traits	Total feed time, min	297.8^B^	65.1	321.1^AB^	61.0	346.1^A^	98.5
	Daily feed intake, kg/d	0.297^C^	0.024	0.324^B^	0.025	0.372^A^	0.061
	Visit feed intake, g	23	5	23	5	25	5
	Meal feed intake, g	33^b^	7	37^b^	6	40^a^	8
	Visit duration, s	76.1	20.7	75.4	21.3	77.5	22.3
	Meal duration, s	147.5^c^	38.8	171.8^a^	40.4	170.1^a^	41.7
	Daily interval between meals, min	165	26	167	23	161	28
	Daily feeding rate, g/min	19.6	3.0	20.3	4.3	21.7	4.0
	Number of meals per day	8.5	3.3	8.4	3	8.7	3.2
	Number of visits per day	12.9	5.9	13.8	5.8	14.5	6.6

BW 19: average body eight at d 19; BW 42: average body eight at d 42; RFI, residual feed intake; FCR, feed conversion ratio. S.D., standard deviation. Different letters in the same row means significant differences between different groups. Capital: *P <* 0.01*;* Lowercase: *P <* 0.05

Phenotypic correlations between feeding behaviors and other traits in different RFI levels group were illustrated in Table 4. Unexpectedly, body weight and body weight gain was positively correlated with DFI in different levels ducks. DFI and MD were observed to be significantly correlated with feeding efficiency in high RFI ducks (*P* < 0.05). Moreover, significant correlation between TFT and feed efficiency was found in both low and high RFI level ducks. For DFR, only correlation with RFI was observed in low RFI level.

**Table 4:** Phenotypic correlation between feeding behavior and other traits in different RFI groups

	Total Feed Time			Daily Feed Intake			Daily Feeding Rate			Between-Meal Pause			Meal Feed Intake			Meal Duration		
RFI Group	H	M	L	H	M	L	H	M	L	H	M	L	H	M	L	H	M	L
BW 19	0	−0.11	0.31	0.19	0.52**	0.33	−0.03	0.24	−0.12	0.14	0.18	0.43*	0.14	0.41*	0.48**	−0.01	0.13	0.01
BW 42	0.21	−0.01	0.32	0.48**	0.79**	0.79**	−0.11	0.32	0.1	0.12	−0.06	0.04	0.25	0.33	0.46**	0.18	0.06	0.08
BWG	0.22	0.05	0.23	0.41*	0.65**	0.73**	−0.10	0.24	0.16	0.03	−0.18	−0.12	0.20	0.15	0.32	0.25	−0.01	0.08
FI	0.41*	−0.02	0.55**	0.77**	0.8**	0.54**	0.16	0.31	0.03	−0.36*	−0.03	0.02	0.18	0.36*	0.26	0.5**	0.08	0.12
RFI	0.36*	0.13	0.38*	0.67**	0.08	−0.03	0.39*	−0.1	−0.04	−0.4*	0.1	−0.05	0.1	0.11	−0.11	0.45**	0.16	0.08
FCR	0.32	−0.09	0.39*	0.35*	0.09	−0.1	0.30	0.04	−0.13	0.10	0.29	0.15	0.08	0.3	0	0.41*	0.15	0.05

BW 19: average body eight at d 19; BW 42: average body eight at d 42; “**“*P* < 0.01;”*” *P* < 0.05. H, M, L mean High RFI group, Middle RFI group and Low RFI group, respectively

### Changes in feeding behavior over time

**Fig. 1:**
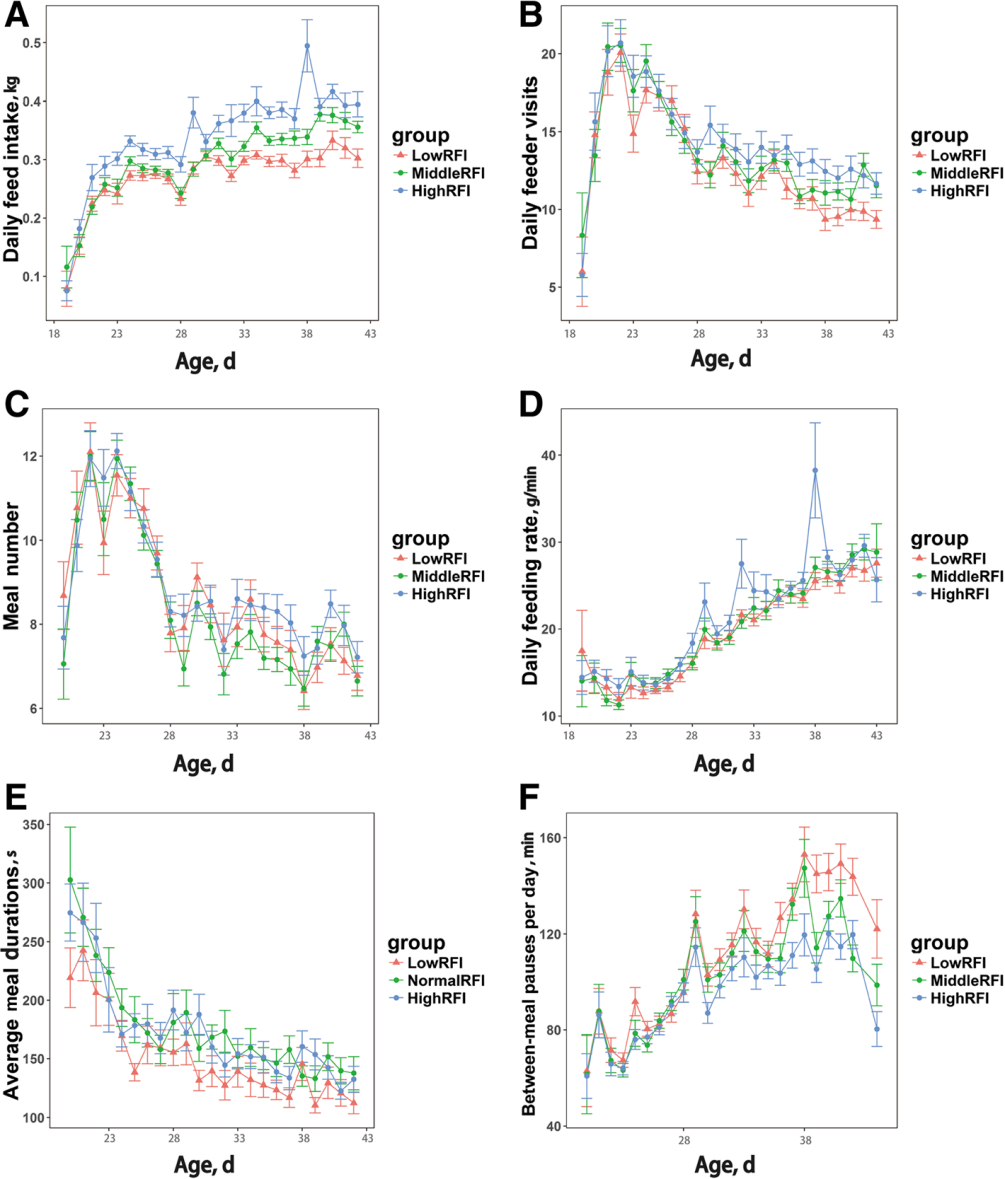
Evolution of feeding behavior traits during the feeding test period. X axis denotes the age (days), and Y axis denotes daily mean of each feeding behavior trait. Error bars are illustrated on the plots. The data from the last day was removed, since ducks are considered to be under stress during this fasting period. Ducks were 19 d of age at the beginning of the experiment. **a** Daily feed intake (**b**) Number of daily visits (**c**) Number of daily meals (**d**) Daily feeding rate (**e**) Daily meal duration (**f**) Between-meal pause

To further understand the relationship between RFI and feeding behavior, changes in feeding behavior over time were investigated and are illustrated in Fig. 1. During the test period, average DFI increased from 0.07 kg to 0.35 kg (Fig. 1a), and the effect of RFI level was significant from the 3^rd^ day to the end of test (*P* < 0.01). The average number of daily visits and meals increased constantly in the early days, then gradually diminished, and finally stabilized (Fig. 1b, c). Obvious differences in daily visits were found among different RFI level from 4 wk. To the end of test, and these differences magnified with age; however, there was no difference in daily meal number. The daily feeding rate increased over the entire test period (Fig. 1d, e); in contrast, meal duration gradually decreased with age. Moreover, between-meal pauses was observed to increase until the 11^th^ day, then was maintained at a relative stable value, and finally, in the between-meal pauses of low RFI ducks were slightly longer than high RFI animals.

### Feeding tendency

**Fig. 2:**
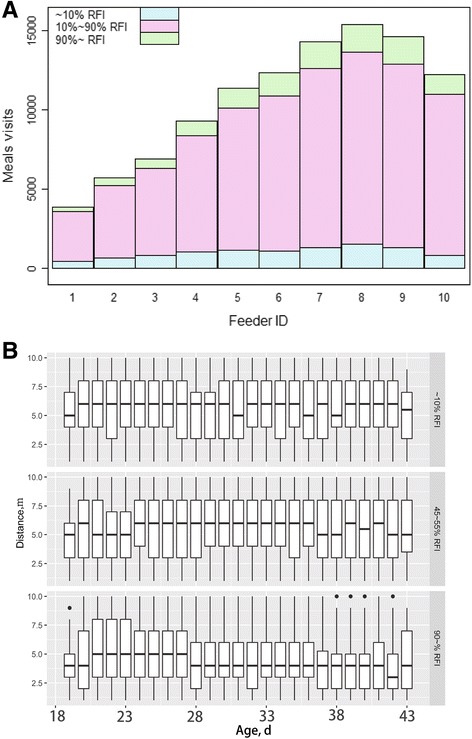
**a** The distribution of visits recorded by feeders. X axis denotes the feeder ID, and Y axis denotes the number of visits. Different colors represent three RFI levels of the ducks, respectively. Feeding machine is linearly layout from number one to ten. The first machine was closest to the gate, and the last one was far away from gate. All of invalid visits were removed. **b** Comparison of duck feeding tendencies in different RFI level groups. X axis represents the age of birds, and Y axis represents the distance between feeder and door. The box plot represents the range of feeding activities at different RFI levels. The data of the last day was removed, because at this fasting time the ducks were considered to be under stress. Variance analysis showed that the machine position that high RFI ducks tended to eat in was significantly lower (i.e. closer to the gate) than other ducks, over the entire time period (*P* < 0.01). The initial bird age was 19 d

Fig. 2a summarizes the records of all the feeders. The machine with the highest number of visits is the eighth feeder which has 15,102 visits, and the feeder with the lowest records was the first one, with 4422 visit records. Ducks in the top 10% RFI level recorded 14% of the total records, and visit records of the lowest 10% RFI animals were 11% of the total. To further understand the feeding tendencies of ducks, the distance to the gate was used to define meal positions (Fig. 2b). The results show that animals were concentrated in middle positions (4 to 5 m from gate) for the first two days. After an adaption time, more ducks preferred to eat at 7 ~ 8 m from the gate; however, high RFI ducks frequently visited feeders at positions of 3 to 4 m from the gate, in which frequency was 1.70 per day, higher than 1.57 per day for low RFI animals. The feeder machines visited by high RFI ducks was closer to gate than for low and middle RFI ducks.

### Distribution of daily feeding time

**Fig. 3:**
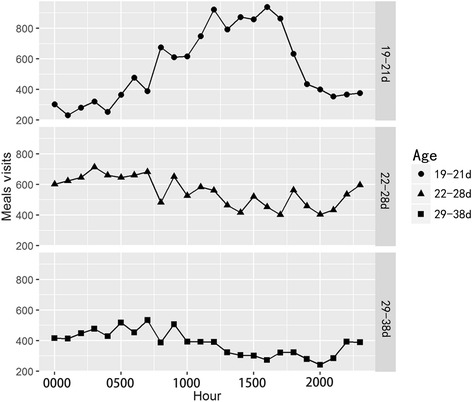
The feeding visit distribution during the day. X axis denotes the time points (hr), Y axis denotes the number of visits. The results for three different periods are illustrated: 19–21 day-old; 22–28 day-old; 29–38 day-old; the initial bird age was 19 d. The number of each RFI group is 33

To investigate the daily feeding time of ducks, the feeding records at different time points every day are summarized in Fig. 3. The results showed that duck feeding was mainly concentrated at noon and in the afternoon during the adaptation period, and reached the highest peak between 1200 h at noon and 1600 h in the afternoon. In the pre-growth period, feeding activity was observed during the entire 24 h period, and the number of visits increased significantly from 2300 h at night to 0500 h in the early morning. Compared to the first 10 d, the number of feeding visits was reduced during the growth period, but the tendency (frequent activities from 2300 h at night to 0700 h in the early morning) was similar to the pre-growth period, and was concentrated from midnight to the following morning.

## Discussion

### Meal determination

Feeding behavior consists of different feeding activities, which are referred to feeder visits. Bley and Bessei [12] found that Pekin ducks exhibited an average of 29 visits per day at 4 wk of age, decreasing to fewer than 15 visits per day at 7 wk of age. Basso et al. [11] obtained a similar trend with 27 visits per day at 3 wk, rapidly decreasing to 15 visits, and then stabilizing. In this study, we found that the number of visits was gradually reduced from an average 25 visits per day at 3 wk of age to 14 visits at 4 wk, and then was stable at about 13 visits until the end of the study at 7 wk (Fig. 1b).

Our results are consistent with the two studies above. Actually a meal is not always equal to one visit, but rather contained a few visits and short relaxation pauses called within-meal pauses for animal relaxing or drinking; frequently, meals are separated by non-feeding intervals called between-meal pauses which are much longer than within-meal pause [11]. Therefore, a meal criterion is needed for grouping different feeding events into defined meals [15, 16]. Howie et al. [17] obtained a meal criterion of 1725 s for Pekin ducks by fitting a truncated log-normal model, and Basso et al. [11] found the meal criterion of 1790 s for Mule ducks using same method; however, Drouilhet et al. [18] estimated the meal criterion as 2208 s in their study in which the feeder entrance had a door. In our study, the log distribution of visit intervals is similar to the trends reported by Howie et al. [15] and Basso et al. [11] (Additional file 2: Figure S2); hence, we used Howie’s method for our data set and calculated a meal criterion of 1,132 s for Pekin ducks. This value is, lower than the meal criterion reported by others, which is likely due to the differences in feeder construction and in duck population. We found that meals consisted on average of 1.7 visits and that the number of meal per day had a similar trend in three RFI-level. Basso et al. [11] and Drouilhet et al. [18] found that the number of meals tended to decrease with age; however, between-meal pauses tended to increase. In boilers, Howie et al. [15] also found same trend. Our results showed that the number of meals tended to decrease with age (from 12 meal/d to 7 meal/d) after feeder adaption time and the between-meal pauses tended to increase (from 60 min/d to 2 h/d more). The results are in agreement with previous studies.

### Feeding tendency at different RFI levels

Feeding tendency is possibly affected by environmental factors, especially under a wide range of farming conditions. These changes may be due to management mechanisms or system structures. For example, sward structure and herbage quality affected cattle’s grazing behavior [19]. Another study showed that the average time cattle spent lying increased with increasing number of concentrate feeders [20]. Funston et al. [21] found different group composition affected cattle feeding tendencies. In ducks, Drouilhet et al. [18] used a feeder with a door closing behind the animals, and reported that this affected the visit intervals. In our study, most ducks tended to eat at the inside feeders which are far away from the gate. However, the innermost feeder was not the best feeding position, since the feeding machine slightly near the center have the highest recorded number of visits (Fig. 2a). This suggests that ducks spontaneously move away from the gate where animals are susceptible to environmental interference. In contrast, the innermost machine is limited by proximity to the fence and this could greatly reduce activity space for ducks. Our results also show that high RFI ducks tend to eat near the gate, where high RFI ducks were more susceptible to outside interface (Fig. 2b), and this always occurs during the entire period. Some studies indicate that selection for feed efficiency might reduce behavioral reactivity [22], and evidence reveals that animals with better feed efficiency have lower fearfulness. Genetic research with mammals and poultry showed that low RFI animals were less active [23, 24]. Drouilhet, et al. [18] reported that selection for low RFI produces mule ducks that are slightly better adapted to human activity, in agreement with a previous study [25]. Compare to their results, our study suggests that high RFI individuals have lower adaptive ability to the new feeding environment. However, there is not enough evidence to determine the clear reason for this effect. Moreover, we found that feeding time points for adaptation during the first 3 d were different than at other time periods (Fig. 3). Ducks mainly were eating at noon and early afternoon during first 3 d while adapting to the feeders. After adaptation, ducks returned to middle of the night eating habits. These behaviors may be improved by feeder structure modification and selection for adaption response.

### Feeding behavior at different RFI levels

The relationship between feeding behavior and feeding efficiency depends on population and species, and there are obvious differences in feeding behavior between mammals and poultry selected for feed efficiency. Studies with cattle reported that high RFI cattle consume more feed, but with similar feeding rates, compared to low RFI animals [26, 27]. For pigs, Young et al. [28] found that low RFI animals had shorter feeding times with a lower DFI compared to the high RFI pigs, and that the feeding rates of low RFI animals were significantly faster than the control group. A similar result was obtained in a hen study; Braastad and Katle [23] found that low RFI birds had shorter feeding times than high RFI birds. Drouilhet et al. [18] found that low RFI mule ducks had significantly lower DFI than high RFI ducks, but no differences were significant between high and low RFI lines for other feeding behavior traits. In this study, we found there was a significant phenotypic correlation between feed efficiency and feeding behaviors except for BMP (Table 2). It indicated that the feeding frequency has a limited impact on feed efficiency, total feeding time and daily feed intake are more critical. The Low RFI ducks spent less feeding time and had a lower daily feed intake than High RFI ducks. However these correlative relationships were not observed in different RFI-level sub-populations (Table 4). Therefore, selection for RFI may change the association between feeding behavior and other traits.

Our results were different with those of Drouilhet, et al. [18]; this discrepancy could be caused by feeder structure and selection of meal criterion. Additionally, when compared day by day, ducks in the three different RFI levels had a similar number of daily visits at 4 wk of age; however, after 10 d of the test period, low RFI ducks had a significantly lower visit number than high RFI ducks, and these differences were magnified with age (Fig. 1b). Over the entire test period, these changes were too microscopic to achieve statistical significance. Our results gave a possibility that selection for RFI might change the unknown mechanisms that underlie control of feeding behavior, but more genetic evidences were needed to support the hypothesis. This showed that selection for feeding behavior is a valuable approach for trait improvement.

## Conclusions

In this study, we selected 358 male Pekin ducks and measured their feeding behavior, feed efficiency traits in order to analyze relationships between feeding behavior and feed efficiency. The results illustrate that the feeding tendency of Pekin ducks is obviously different at different RFI levels, and that high RFI ducks prefer the feeders closed to side barriers. The results also suggests that certain feeding behaviors have the potential to improve other economic traits with an improvement in feed efficiency. Further research will focus on genetic factors to gain insight into the relationship between feeding behavior and feed efficiency.

## Additional files

Additional file 1: Figure S1. Diagram depicting the pen organization and structure. (PDF 32 kb)

Additional file 2: Figure S2. Density distribution of log e transformed pauses between visits to feeder. X represents the meal criterion. (PDF 203 kb)

## Abbreviations

BMP: Between-meal pauses
BW: Body weight
BWG: Body weight gain
DFI: Daily feed intake
DFR: Daily feeding rate
FCR: Feed conversion rate
FI: Feed intake
MD: Meal duration
MFI: Feed intake per meal
RFI: Residual feed intake
RFID: Radio Frequency Identification
TFT: Total feeding time
