# Supplementary figures and images for: Systematic analysis of feeding behaviors and their effects on feed efficiency in Pekin ducks
Source: J Anim Sci Biotechnol. 2017 Nov 1;8:81. doi: 10.1186/s40104-017-0212-2 (PMC5664582; doi:10.1186/s40104-017-0212-2)

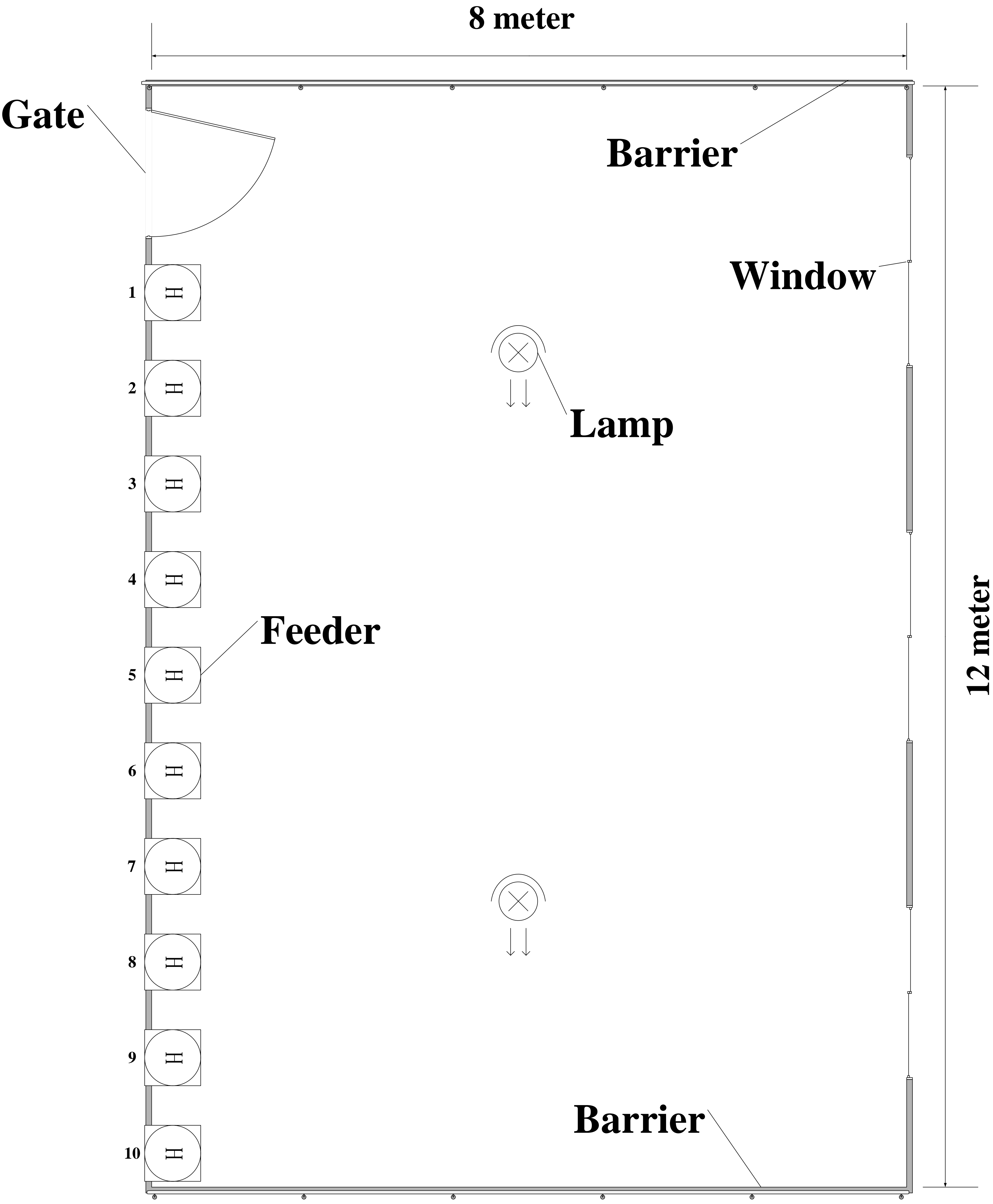

Supplement: Supplementary file 1 — Diagram depicting the pen organization and structure. (PDF 32 kb) [file 40104_2017_212_MOESM1_ESM.pdf]

# The distribution of log-transformed visits intervals

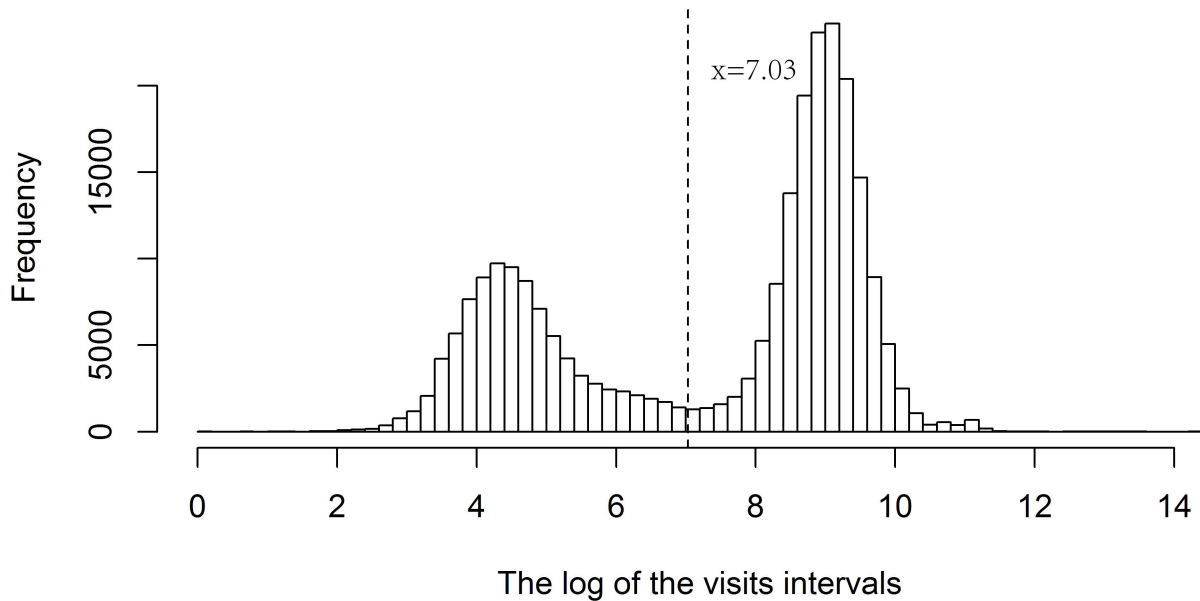

Supplement: Supplementary file 2 — Density distribution of log e transformed pauses between visits to feeder. X represents the meal criterion. (PDF 203 kb) [file 40104_2017_212_MOESM2_ESM.pdf]
